# A latent factor framework to organize regulatory and metabolic programs inferred from scRNA-seq

**DOI:** 10.1093/bioadv/vbag185

**Published:** 2026-06-30

**Authors:** Chiara Napoli, Francesco Bardozzo, Suraj Verma, Le Minh Thao Doan, Pierpaolo Fiore, Carmen Faggiano, Claudio Angione, Annalisa Occhipinti, Roberto Tagliaferri

**Affiliations:** NeuroneLab—Department of Management and Innovation Systems (DISA-MIS), University of Salerno, Fisciano, SA 84084, Italy; Department of Biochemical Sciences “A. Rossi Fanelli”, Sapienza University of Rome, Rome, RM 00185, Italy; NeuroneLab—Department of Management and Innovation Systems (DISA-MIS), University of Salerno, Fisciano, SA 84084, Italy; NAIR Center (Navarra Artificial Intelligence Research Centre), Calle del Sadar, Pamplona, 31006, Spain; School of Computing, Engineering and Digital Technologies, Teesside University, Middlesbrough, TS1 3BX, United Kingdom; School of Computing, Engineering and Digital Technologies, Teesside University, Middlesbrough, TS1 3BX, United Kingdom; NeuroneLab—Department of Management and Innovation Systems (DISA-MIS), University of Salerno, Fisciano, SA 84084, Italy; NeuroneLab—Department of Management and Innovation Systems (DISA-MIS), University of Salerno, Fisciano, SA 84084, Italy; School of Computing, Engineering and Digital Technologies, Teesside University, Middlesbrough, TS1 3BX, United Kingdom; School of Computing, Engineering and Digital Technologies, Teesside University, Middlesbrough, TS1 3BX, United Kingdom; NeuroneLab—Department of Management and Innovation Systems (DISA-MIS), University of Salerno, Fisciano, SA 84084, Italy

## Abstract

**Motivation:**

Single-cell RNA sequencing enables high-resolution characterization of transcriptional heterogeneity, but provides only a partial view of the regulatory and metabolic processes associated with cellular states. Several computational methods infer transcription factor (TF) activity and metabolic features directly from RNA, yielding complementary functional representations of cellular organisation.

**Results:**

Here, we use a latent factor organisational strategy to jointly model four transcriptome-derived functional projections, namely gene expression, TF regulon activity, metabolite-level features and predicted metabolic fluxes. Although all layers originate from the same measurement, each captures distinct regulatory or metabolic programs. The resulting latent space organizes these inferred programs into coordinated axes of variation guided by complementary regulatory and metabolic constraints, facilitating functional interpretation beyond gene expression alone. When applied to a breast cancer cell line dataset, the proposed framework identifies distinct functional programs, including proliferative, oxidative-metabolic and stress-associated axes, that are only partially resolved in RNA-only analyses of this dataset. Overall, our results suggest that regulatory and metabolic programs inferred from scRNA-seq can be structured into an interpretable latent representation, supporting a more coherent functional characterization of cellular states from transcriptome-derived functional projections.

## 1 Introduction

Single-cell RNA sequencing (scRNA-seq) enables high-resolution analysis of cellular heterogeneity and has motivated the development of widely adopted computational frameworks such as Seurat (Satija *et al.* 2015), Scanpy (Wolf *et al.* 2018), and Bioconductor-based workflows (Gentleman *et al.* 2004). These approaches have established robust standards for transcriptomic analysis; however, they primarily describe variation at the level of gene expression and therefore provide only a partial representation of the regulatory and metabolic processes underlying cellular states.

In recent years, two complementary methodological directions have emerged. On one hand, advances in multi-omic technologies have enabled the direct measurement of multiple molecular layers (Hayes *et al.* 2024), motivating the development of integrative models designed to jointly analyse matched experimental modalities (Baião *et al.* 2025, Zhang *et al.* 2025). This has led to a range of statistical and machine-learning frameworks aimed at capturing shared and modality-specific sources of variation, including probabilistic and deep learning approaches such as MOFA+ (Argelaguet *et al.* 2018, 2020), scMM (Minoura *et al.* 2021), and MultiVI (Ashuach *et al.* 2023). These methods are explicitly designed for multi-omic measurements and focus on disentangling biological signals across experimentally distinct data types. Among these approaches, probabilistic factor models such as MOFA+ provide an interpretable framework for identifying latent sources of variation shared across heterogeneous molecular layers, making them particularly suitable for exploring coordinated biological programs. These developments provide a useful conceptual basis for modelling coordinated variation across multiple data representations. In the present context, however, the views are transcriptome-derived functional representations rather than experimentally independent modalities. This makes interpretability of the shared latent structure particularly important: the goal is not to disentangle separate molecular assays, but to organize continuous regulatory and metabolic projections of the same cells into coordinated axes of variation. MOFA+ is well suited to this objective because it provides an interpretable probabilistic framework for identifying coordinated sources of variation across heterogeneous continuous views, with view-specific loadings that facilitate direct biological interpretation of the latent factors.

On the other hand, functional inference methods enable the derivation of additional biological representations directly from scRNA-seq data, estimating higher-order regulatory and metabolic activities that complement raw gene expression (Tzec-Interián *et al.* 2025). Examples include transcription factor (TF) regulon activity inferred using SCENIC (Van de Sande *et al.* 2020, Kumar *et al.* 2021) and metabolic flux predictions obtained with scFEA (Alghamdi *et al.* 2021). These inferred representations can be interpreted as biologically constrained functional projections of the transcriptome, summarizing regulatory and metabolic aspects of cellular variation that may be less explicit when cells are represented only in gene expression space. While recent studies have shown that combining multiple inferred views can improve interpretability in the absence of matched multi-omic measurements (Lapuente-Santana *et al.* 2024), these functional representations are often analysed independently, limiting the assessment of their coordinated variation within individual cells. As a consequence, it remains unclear whether regulatory and metabolic signals inferred from the same transcriptome support coherent cellular programs when considered jointly at the single-cell level.

In this study, we bridge these two directions by addressing whether regulatory and metabolic programs inferred from scRNA-seq can be organized into a structured latent representation that clarifies cellular states beyond gene expression alone. Building upon latent factor models originally developed for multi-omic data integration (MOFA+), we repurpose them to organize multiple functional projections inferred from a single transcriptomic modality. Four transcriptome-derived representations, scRNA expression, TF regulon activity, metabolite-level features and predicted metabolic fluxes, are jointly modelled within a shared latent space. Although all views originate from the same scRNA-seq measurement, each captures a distinct regulatory or metabolic aspect of cellular organization. Rather than increasing the amount of molecular information, our framework aims to organize complementary transcriptome-derived signals into coordinated functional axes that facilitate the biological interpretation of cellular states. This perspective uses multi-view modelling as a strategy for structuring heterogeneous functional representations of the transcriptome, rather than as a substitute for matched multi-omic integration.

In Section 2, we describe the construction of the four transcriptome-derived functional layers and the multi-view modelling strategy. In Section 3, we apply the framework to HER2+ breast cancer cell lines, where the multi-view latent representation reveals reproducible axes of variation associated with proliferative, oxidative-metabolic and stress-related programs. Additional analyses on Triple-Negative, Luminal A and Luminal B subtypes are provided in the Supplementary Information, available as supplementary data at *Bioinformatics Advances* online, supporting the applicability of the organizational strategy across distinct molecular contexts.

## 2 Methods

### 2.1 Framework overview

**Figure 1:**
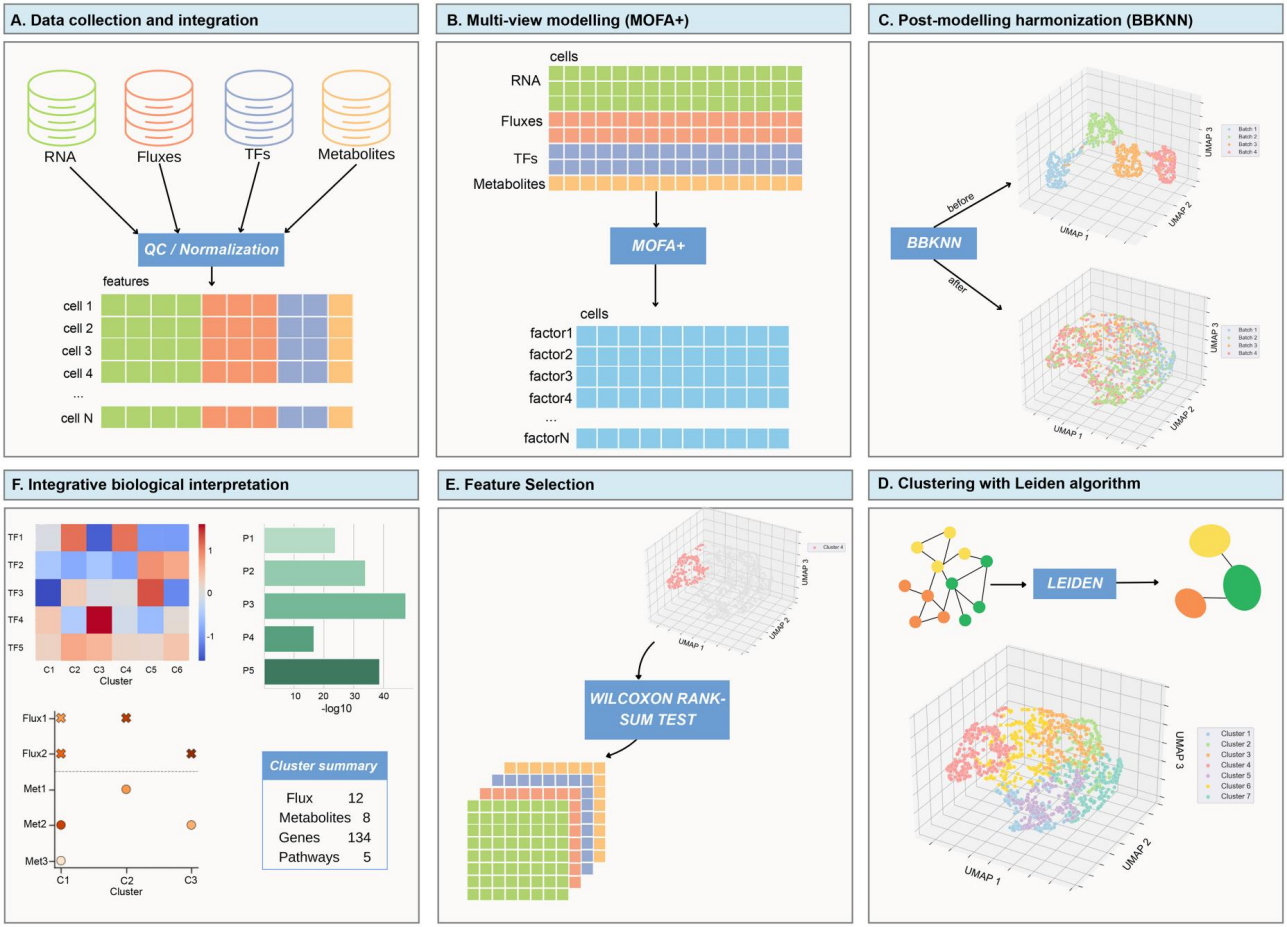
Overview of the functional multi-view modelling framework. (A) Data collection and integration. Transcriptome profiles at single-cell resolution are used to infer four aligned functional layers per cell: scRNA expression, TF regulon activity (pySCENIC), metabolite-level features and metabolic fluxes (scFEA). All matrices are normalized and standardized prior to modelling. (B) Functional multi-view modelling with MOFA+. The four matrices are jointly modelled using a probabilistic latent factor model to learn shared and view-specific components capturing transcriptional, regulatory and metabolic variability. (C) Post-modelling harmonization. A BBKNN neighbourhood graph is constructed using cell-line identity as a grouping variable to balance local neighbourhood composition across cell lines without altering the global latent structure. (D) Clustering with the Leiden algorithm. Cells are clustered in the harmonized latent space to define coherent functional states. (E) Feature selection. Differential analysis identifies cluster-specific markers independently within each functional layer. (F) Integrative biological interpretation. Marker genes, TFs, metabolites and fluxes are integrated across layers to derive composite functional profiles per cluster.

This section outlines a six-step analytical workflow for the organization and downstream analysis of complementary functional layers inferred from scRNA-seq data. As illustrated in Fig. 1, the workflow consists of: (i) data collection and processing, including quality control, normalization and inference of regulatory and metabolic features from transcriptomic profiles (Section 2.2); (ii) latent factor modelling of aligned functional layers (Section 2.3); (iii) post-modelling harmonization of the latent representation (Section 2.4); (iv) clustering analysis performed on the harmonized embedding (Section 2.5); (v) cluster-specific feature selection across transcriptomic, regulatory and metabolic layers (Section 2.6); and (vi) integrative biological interpretation based on the combined analysis of the selected features (Section 2.7).

Cellular data were represented in a multi-view configuration. Four aligned data views were constructed for each cell, capturing distinct aspects of molecular function: scRNA expression, TF regulon activity, predicted metabolite-level features and predicted metabolic fluxes. In this setup, all matrices shared identical cell ordering to ensure one-to-one correspondence across views. Although each layer was inferred from the same transcriptomic input, the views described complementary transcriptional, regulatory and metabolic dimensions of cellular identity and were therefore treated as distinct functional views within a multi-view modelling framework.

Importantly, these views correspond to different biological entities (genes, TF regulons, metabolites and metabolic reactions) and therefore occupy distinct feature spaces. The modelling strategy must therefore accommodate heterogeneous feature representations while preserving coordinated variation across cells.

Multi-view modelling resulted in a unified latent representation capturing coordinated transcriptional, regulatory and metabolic variation across cells. This representation was used as the basis for downstream harmonization, clustering and feature-level analyses, as described in the following sections.

As a benchmark, a conventional scRNA-seq analysis pipeline was implemented using Scanpy (*v*1.10.3), including dimensionality reduction, clustering, differential gene expression analysis and gene set enrichment analysis. This scRNA-only workflow was used as the primary baseline to quantify the added value of functional multi-view modelling relative to standard transcriptome-based analyses.

In addition, scVI (Lopez *et al.* 2018) was evaluated as a deep generative transcriptomic baseline to assess whether the multi-view latent representation provides complementary biological organization beyond a transcriptome-only embedding.

Cell-cycle scores for S and G2M phases were computed using canonical gene sets via scanpy.tl.score_genes_cell_cycle (Tirosh *et al.* 2016). These scores were not regressed out from the expression matrix. In the present cancer setting, proliferation-associated transcriptional programs represent a major axis of regulatory and metabolic variation, with proliferative heterogeneity often linked to metabolic adaptation, including shifts between glycolytic and oxidative metabolism. By preserving proliferative variation, the multi-view modelling framework can resolve how proliferative states coordinate with regulatory and metabolic programs across the latent space, providing a more complete functional characterization of tumour heterogeneity that would be obscured by cell-cycle regression.

### 2.2 Data collection and integration

We analysed the scRNA-seq dataset published by Gambardella *et al.* (2022), publicly available in the Gene Expression Omnibus (GEO) repository under the accession code *GSE173634*. This dataset profiles multiple breast cancer cell lines spanning four molecular subtypes, including HER2+, Triple-Negative, Luminal A and Luminal B.

We focused on the HER2+ subset as the main case study due to its clinical relevance and heterogeneity (4324 cells across five cell lines). The broad applicability of the framework is further supported by additional analyses performed on other breast cancer subtypes, which are reported in the Supplementary Information, available as supplementary data at *Bioinformatics Advances* online.

After standard quality control and normalization, we derived four aligned functional layers for each cell.

For the RNA layer, 3386 highly variable genes (HVGs) were selected using Scanpy with mean-dispersion thresholds (min_mean = 0.0125, max_mean = 3, min_disp = 0.5). Both the scRNA-only baseline workflow and all cross-layer comparisons were conducted using this HVGs-restricted expression matrix.

Functional inference of regulatory and metabolic layers was performed on the full set of expressed genes, as required for accurate reconstruction of gene regulatory networks and estimation of metabolic activity.

TF regulon activity was inferred using pySCENIC v0.12.0 in a reproducible Docker environment (AertsLab/pyscenic: 0.12.0), following the standard three-step workflow consisting of gene regulatory network inference with GRNBoost2, motif enrichment with RcisTarget, and regulon activity scoring with AUCell. This resulted in activity scores for 79 TF regulons, which constituted the regulatory view used for multi-view modelling.

Metabolic features were inferred using the scFEA framework (Alghamdi *et al.* 2021, Zhang *et al.* 2023), which estimates relative metabolite abundances and reaction-level fluxes by projecting single-cell transcriptomic profiles onto a genome-scale metabolic network. The *M*171 human metabolic map was used, comprising 168 metabolic reactions organized into 22 supermodules and 70 intermediate metabolites.

Cellular metabolism is modelled under two core assumptions: (i) steady-state mass balance, such that the total influx and efflux of each metabolite are equal, and (ii) reaction fluxes are nonlinear functions of the expression levels of enzymes associated with each metabolic module. The model was trained for 100 epochs on scRNA-seq data to infer per-cell metabolic reaction fluxes and relative metabolite abundances consistent with network stoichiometry and mass-balance constraints.

All views were *z*-score scaled across cells prior to MOFA+ training to place features on a comparable scale. For differential analyses, however, layer-specific tests were performed on view-native values (e.g. raw AUCell scores and unstandardized scFEA outputs), as described in Section 2.6.

### 2.3 Multi-view modelling

Multi-view modelling was performed using MOFA+ (v1.16.0), a probabilistic factor analysis method designed to jointly model multiple functional layers (Fig. 1B) (Argelaguet *et al.* 2018). The four aligned matrices were modelled jointly using Gaussian likelihoods. MOFA+ identifies latent factors that capture shared and view-specific sources of variation, thereby summarizing coordinated biological signals across views.

MOFA+ is particularly suited for this framework because it can integrate heterogeneous views defined on distinct feature spaces through a shared latent representation, without requiring a common set of features across views.

A model with 10 factors was selected after assessing model convergence and the variance explained across factors. The variance explained decreases progressively, with diminishing returns from additional factors beyond factor 10, supporting the selected model (Fig. 1, available as supplementary data at *Bioinformatics Advances* online; Table 6, available as supplementary data at *Bioinformatics Advances* online).

This unified low-dimensional embedding provided the basis for downstream harmonization, clustering, and integrative interpretation.

### 2.4 Post-modelling harmonization

MOFA+ factors were imported into Python as an AnnData object (Virshup *et al.* 2024) and used as the low-dimensional representation (use_rep=“X_mofa”). Although MOFA+ captures global biological structure, it does not explicitly define local neighbourhood relationships. To obtain a coherent manifold and prevent cell-line identity from dominating kNN graph construction, we applied BBKNN using cell_line as the grouping variable.

BBKNN rebalances only the local neighbour graph, without altering the underlying MOFA+ latent embedding, thereby preserving global latent structure while reducing cell-line-driven neighbourhood imbalance and enabling consistent clustering and visualization (Becht *et al.* 2018). This step reduces the influence of cell-line-specific neighbourhood imbalance and supports clustering of shared functional structure across cell lines.

### 2.5 Cluster analysis

As illustrated in Fig. 1D, Leiden clustering (Traag *et al.* 2019) was performed on the BBKNN-corrected neighbourhood graph derived from the MOFA+ latent factors (use_rep=“X_mofa”). Graph-based community detection methods are widely adopted in scRNA-seq analysis as a best-practice approach for identifying cellular populations from low-dimensional representations, with Leiden recommended over Louvain due to its ability to produce well-connected communities and improved computational efficiency (Heumos *et al.* 2023).

Clustering was performed across a range of resolutions (0.4–1.0), and robustness was assessed using a subsampling-based adjusted Rand index (ARI). A resolution of 0.6 was selected as a trade-off between clustering stability and granularity (ARI 0.59±0.06), avoiding overly coarse partitions at lower resolutions and fragmentation at higher resolutions.

Clustering robustness was evaluated quantitatively through repeated 80% cell subsampling, providing a stability-based assessment of the latent representation across subsampled datasets.

Silhouette-based metrics were not used, as they are known to be poorly suited for graph-based embeddings and integrated single-cell representations (Rautenstrauch and Ohler 2026).

To verify that the resulting clusters reflected structured biological variation rather than residual cell-line-driven effects, MOFA+ factors enriched in each cluster and their dominant feature loadings were examined. Consistency between factor-feature profiles and cluster-specific markers identified independently within each functional layer supported the biological coherence of the cluster structure.

### 2.6 Feature selection

Cluster-specific features (Fig. 1E) were identified independently in each functional layer by comparing each cluster against all remaining cells using the Wilcoxon rank-sum test (Heumos *et al.* 2023) (Scanpy implementation). Multiple testing corrections were applied using the Benjamini-Hochberg procedure to control the false discovery rate (FDR).

For the scRNA layer, differential expression analysis was performed on log-normalized counts. Marker genes were defined as those with adjusted *P* value <.05 and log fold-change >0.5.

For TF regulon activities, differential analysis was performed using the same Wilcoxon-based framework. AUCell scores were used in their original scale (not *z*-scored); because they are bounded between 0 and 1 and reflect relative activity, log fold-changes are not meaningful, and mean differences were reported as effect sizes.

For metabolite features and metabolic fluxes, differential abundance analysis was performed on the original (non-*z*-scored) values. Since these quantities can take both positive and negative values and are not defined on a multiplicative scale, effect sizes were reported as mean differences between clusters.

Features were considered upregulated when their mean value in a cluster exceeded that of the remaining cells while meeting the significance threshold. This provided a uniform testing procedure across layers while keeping the interpretation of each view consistent with its native scale.

### 2.7 Integrative biological interpretation

Functional interpretation at the cluster level was performed by integrating marker features and pathway enrichments across the four layers.

For scRNA expression and TF regulon activity, significant marker genes and regulon targets were subjected to gene set enrichment analysis using GSEApy (Enrichr module) (Fang *et al.* 2023) against the Gene Ontology (2025 release) (Ashburner *et al.* 2000, Cherry *et al.* 2023) database, including the Biological Process (BP), Molecular Function (MF), and Cellular Component (CC) categories.

For metabolite features and metabolic fluxes, significant markers were first mapped to their corresponding metabolic reactions and associated genes using the gene-reaction associations provided in the scFEA reference metabolic model. The resulting gene sets were then used for enrichment analysis in the same pathway libraries.

This gene-centric strategy enabled the comparison of transcriptional, regulatory and metabolic signals within a shared functional annotation space. By mapping all layers to gene-level representations and analysing enrichment profiles jointly, the framework allows the identification of functional programs supported by concordant signals across multiple inferred biological layers.

The resulting enrichment profiles revealed both shared biological processes and layer-specific regulatory or metabolic adaptations.

## 3 Results

### 3.1 scRNA-only baseline analysis

**Figure 2:**
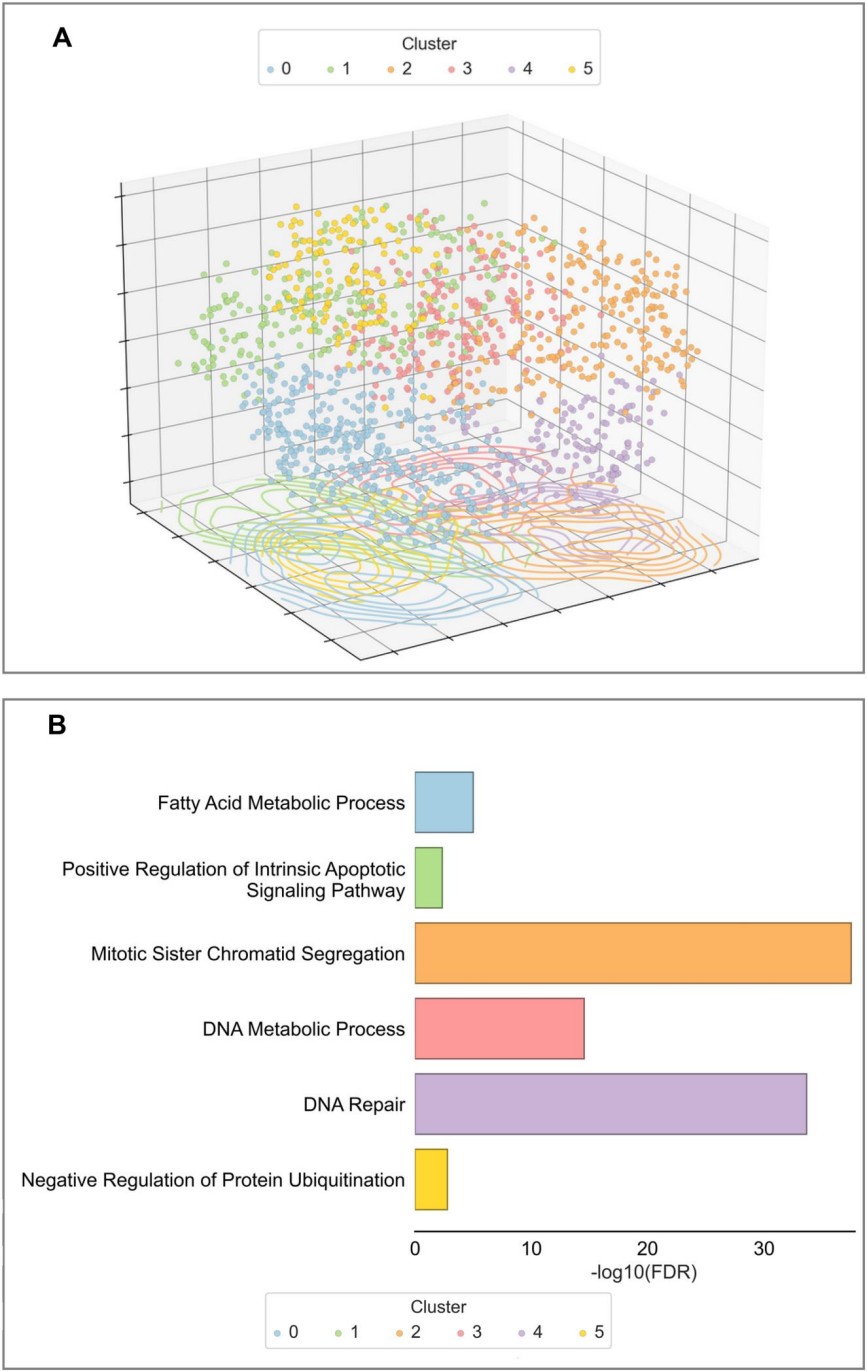
scRNA-only baseline analysis. (A) 3D UMAP embedding of HVGs, with cells coloured by Leiden clusters with 0.6 resolution (six clusters). Density iso-contours on the base plane visualize cluster distribution. A random 30% subsampling was used solely for the 3D visualization to reduce overplotting, without affecting the cluster distribution. (B) Top enriched GO BP terms associated with up-regulated genes in each cluster, ranked by significance ($-{\;\text{log}\;}_{10}$FDR).

We first constructed an scRNA-only baseline using HVGs for dimensionality reduction, batch-aware neighbour graph construction (BBKNN) and Leiden clustering (resolution = 0.6; Fig. 2A). This analysis identified six transcriptionally defined clusters whose marker genes mapped to heterogeneous and partially overlapping biological processes (Fig. 2B). Consistent with previous observations in HER2+ breast cancer models, one cluster was characterized by strong enrichment for proliferation-associated programs, including mitotic spindle organization, sister chromatid segregation and DNA replication (Gambardella *et al.* 2022). However, these pathways did not fully account for the remaining variability in the RNA-only manifold. Beyond a dominant proliferative program, the remaining clusters were characterized by weaker and partially overlapping signatures—including fatty-acid metabolism, vesicle-mediated transport, ubiquitin-dependent regulation, cytokine signalling and apoptotic processes—resulting in limited separation of non-proliferative functional states (Fig. 2B). This baseline provided a reference for comparison with the integrated multi-view analysis.

### 3.2 Integrated multi-view analysis

**Figure 3:**
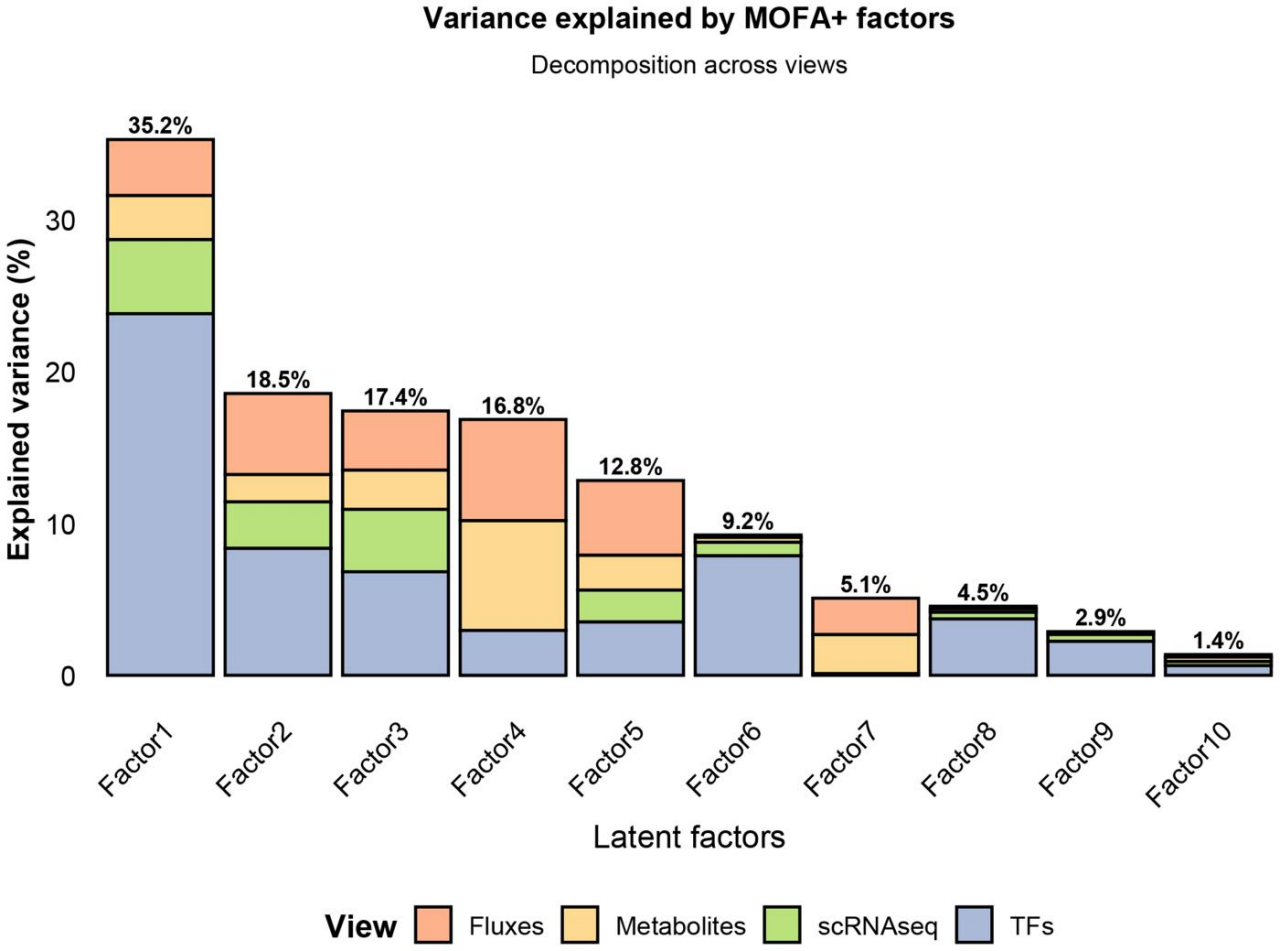
Variance explained by MOFA+ latent factors across functional layers. Each bar represents a latent factor, with stacked colours indicating the proportion of structured variance captured in each functional view. Early factors capture coordinated sources of variation supported across multiple layers, whereas later factors explain smaller fractions of variance and are increasingly dominated by individual views, reflecting more view-specific functional contributions.

**Figure 4:**
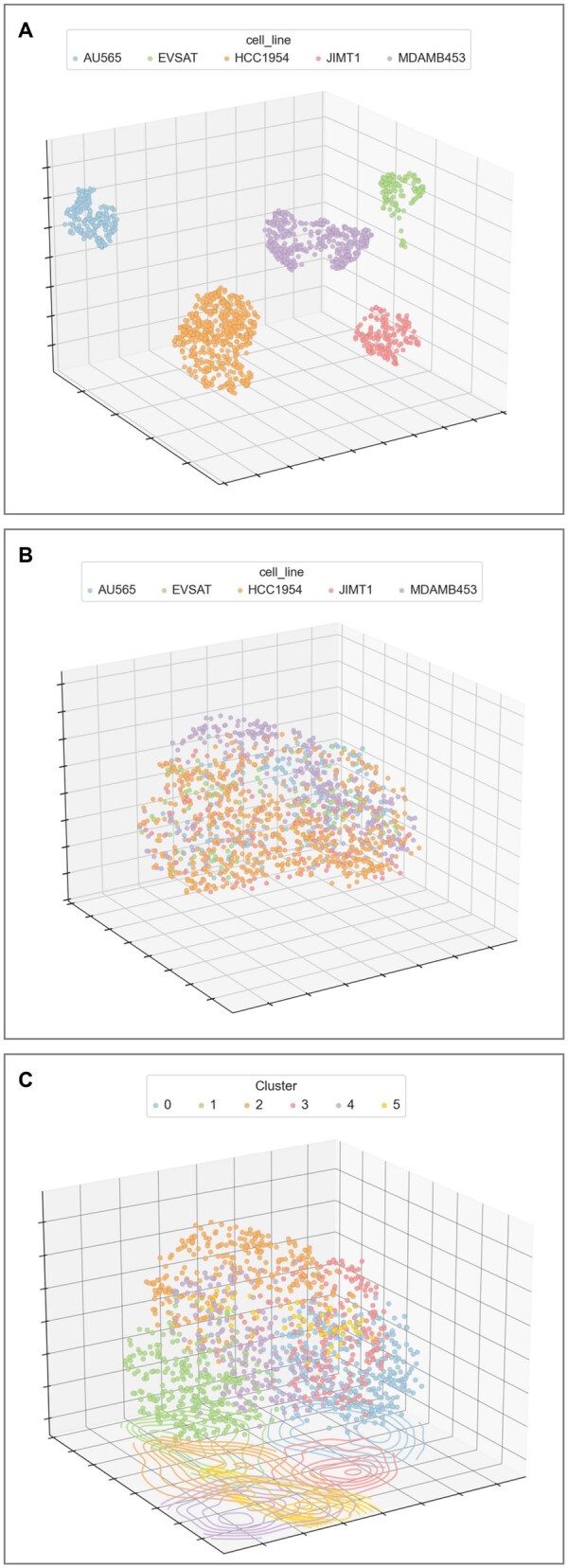
3D UMAP embedding of MOFA+ factors before and after batch correction, and resulting Leiden clusters. (A) UMAP embedding computed from the MOFA+ factors, coloured by cell-line identity. (B) UMAP after BBKNN correction, showing alignment of different cell lines in a shared manifold. (C) Leiden clustering (resolution = 0.6) performed on the BBKNN graph. Density iso-contours highlight the spatial organization of cluster distributions. All 3D UMAPs are shown using a 0.3 subsampling rate for visualization purposes.

To move beyond transcriptome-only analyses, we jointly integrated four RNA-derived functional layers using MOFA+ (Argelaguet *et al.* 2020), a probabilistic factor model capturing shared and view-specific sources of variation across single cells. The resulting latent factors provided a shared low-dimensional embedding of cellular states and were used as the basis for downstream visualization, neighbourhood graph construction and clustering analyses (Fig. 4A). A model with 10 latent factors showed stable convergence and explained complementary fractions of structured variance across layers (Fig. 3). TF regulon activity accounted for the largest share (59.8%), followed by metabolic fluxes (28.2%), metabolite-level features (21.7%), and RNA expression (16.7%). These values represent view-specific reconstruction performance (*R*^2^) rather than total biological variance, and should be interpreted comparatively rather than as absolute measures of information content.

The variance explained (*R*^2^) by MOFA+ factors reflects view-specific reconstruction performance within the latent model, rather than independent information content, since all four layers originate from the same transcriptomic measurement. Differences in *R*^2^ across views are therefore informative about how each inference method structures transcriptomic covariance: TF regulon activity shows the highest reconstruction efficiency because AUCell scores preserve much of the underlying transcriptional covariance structure, whereas metabolic features show lower values reflecting the additional biochemical constraints imposed by the scFEA network model. Rather than indicating redundancy, these differences suggest that each functional layer constrains the latent space in a distinct way, and that their joint modelling enables the resolution of regulatory-metabolic coordination that would not be apparent from any single layer alone.

The MOFA+ embedding was further harmonized using BBKNN to rebalance nearest-neighbour relationships across cell lines while preserving global latent structure (Fig. 4B). Leiden clustering (resolution = 0.6, Fig. 4C) on the BBKNN graph yielded six stable clusters (ARI: 0.59±0.06 under repeated 80% cell subsampling).

Inspection of latent factor loadings showed that clusters were associated with distinct combinations of transcriptional, regulatory and metabolic signals, rather than being driven by a single dominant transcriptional axis. Factors enriched for proliferation-associated features (e.g. Factors 6, 8, and 9) showed strong loadings on canonical cell-cycle regulators, whereas factors enriched for metabolic and stress-related features (e.g. Factors 1, 2, and 5) were dominated by oxidative, inflammatory, or lipid-associated signatures.

### 3.3 Comparison with transcriptome-only representation learning

To assess whether the observed functional organization could be recovered from transcriptomic information alone, we compared the MOFA+ multi-view embedding with a representation learned using the scVI deep generative model (Lopez *et al.* 2018). scVI was applied to the same HVG-restricted expression matrix used in the RNA-only baseline analysis, and the resulting latent representation was processed using the same downstream workflow (BBKNN neighbour graph construction and Leiden clustering).

Clustering robustness was evaluated using the same subsampling-based adjusted Rand index (ARI) framework described above. The scVI embedding showed higher clustering stability than the MOFA+ representation (Fig. 3, available as supplementary data at *Bioinformatics Advances* online), reflecting the fact that scVI optimizes a transcriptome-specific generative model.

**Figure 5:**
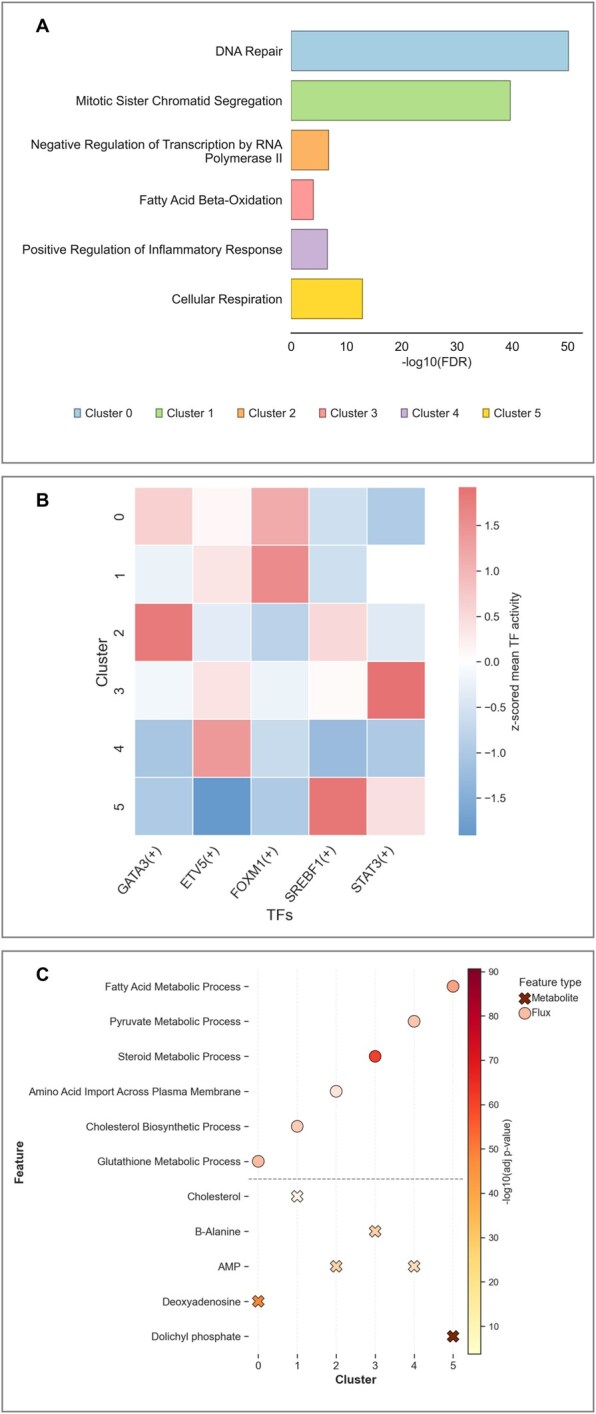
Functional characterization of the integrated clusters across transcriptomic, regulatory and metabolic layers. (A) Top GO BP terms enriched among cluster-specific RNA markers. (B) Cluster-specific TF activity patterns inferred from AUCell regulon scores (scaled mean activity per cluster). (C) Metabolic signatures associated with each cluster, derived from predicted reaction-level fluxes (circles) and inferred metabolite-level features (crosses). Colour indicates statistical significance ($-{\;\text{log}\;}_{10}$FDR), and symbol size reflects effect magnitude.

However, inspection of cluster-associated functional signatures revealed that the scVI representation primarily organized cells along transcriptional gradients dominated by proliferation-related programs, similar to the RNA-only baseline. In contrast, the multi-view MOFA+ representation captured coordinated regulatory and metabolic signals that separated proliferative, oxidative-metabolic and stress-adaptive programs across clusters (Fig. 5).

A quantitative comparison of clustering robustness and RNA-layer interpretability across RNA-only, scVI and multi-view MOFA+ representations is summarized in Table 5, available as supplementary data at *Bioinformatics Advances* online. While scVI provided the most stable clustering (ARI =0.766±0.060), the multi-view MOFA+ representation yielded the highest pathway cluster-specificity (0.784 vs. 0.734 for scVI and 0.708 for RNA-only) and recovered a larger number of cluster-associated RNA GO Biological Process terms (583 vs. 380 for scVI and 394 for RNA-only).

These results indicate that while transcriptome-only representation learning methods can provide robust embeddings of transcriptional variation, integrating regulatory and metabolic functional projections enables a more structured functional interpretation of cellular states.

To quantify cross-view concordance, we computed pairwise RV coefficients between functional layers (Fig. 5, available as supplementary data at *Bioinformatics Advances* online). Regulatory activity showed strong association with RNA expression (RV =0.75), while metabolic views displayed moderate but significant concordance (RV ranging from 0.24 to 0.42). Importantly, after residualizing the dominant RNA component, TF-metabolite and TF-flux relationships remained substantial (RV =0.55 and 0.59, respectively), indicating that coordinated structure across inferred layers is not solely driven by transcriptional variation.

### 3.4 Functional structure recovered by multi-view modelling

The multi-view modelling framework was not designed to increase the number of clusters; rather, it reorganized a comparably granular partition (six clusters at resolution 0.6) into functionally coherent axes supported across regulatory and metabolic layers. This indicates that major biological subdivisions are already encoded within the transcriptome, but may remain only partially resolved when variation is analysed at the level of gene expression alone.

In the multi-view latent representation, cellular heterogeneity was organized into three broad functional modules rather than along a single dominant proliferative gradient (Fig. 5A–C), each supported by internally consistent transcriptional, regulatory, and metabolic signatures.

#### 3.4.1 Proliferative-biosynthetic module (clusters 0 and 1)

Two clusters displayed distinct but related proliferation-associated programs. One cluster was characterized by enrichment of DNA replication, DNA damage response and homologous recombination pathways, together with transcriptional signatures of chromosomal segregation and mitotic spindle organization, and increased glycolytic and ATP-associated metabolic fluxes. The second proliferative cluster showed a mitotic program dominated by spindle organization and sister chromatid segregation, combined with elevated sterol and cholesterol biosynthesis and associated metabolic pathways. In RNA-only embeddings, these signals were partially conflated into a single dominant proliferative cluster, whereas the multi-view latent representation separated them into two coherent programs supported by concordant transcriptional, regulatory, and metabolic features.

#### 3.4.2 Oxidative-metabolic module (clusters 2, 3, and 5)

A second group of clusters exhibited reduced cell-cycle activity and enrichment for oxidative and metabolic programs. These states were characterized by coordinated downregulation of DNA replication and mitotic processes, together with increased amino-acid transport, fatty-acid β-oxidation, monocarboxylate metabolism and oxidative phosphorylation. In RNA-only clustering, these programs were weakly resolved, but emerged as a coherent oxidative-metabolic axis in the integrated latent space supported by consistent regulatory and metabolic signatures.

#### 3.4.3 Stress-adaptive module (cluster 4)

The remaining cluster was associated with stress-adaptive regulatory and metabolic programs. This state showed transcriptional and metabolic signatures linked to inflammatory and immune-related responses, antigen processing and presentation, purine and nucleotide metabolism, and respiration-associated metabolic shifts. These stress-adaptive states were poorly delineated in RNA-only embeddings but became clearly structured when regulatory and metabolic constraints were jointly modelled.

### 3.5 Effects on RNA-level interpretability

We compared RNA-layer GO BP enrichments obtained under RNA-only and integrated cluster definitions. Focusing on up-regulated pathways, the integrated clustering recovered a larger number of significant RNA pathways than the RNA-only analysis (583 vs. 394), retaining most RNA-only pathways (310 shared) while identifying additional pathways uniquely detected under the integrated clustering (273).

To quantify interpretability, we measured *cluster-specificity*, defined as the fraction of significant RNA pathways uniquely associated with a single cluster. Up-regulated RNA pathways showed higher cluster-specificity under the integrated clustering than under the RNA-only clustering (0.784 vs. 0.708), despite both analyses using the same transcriptomic data and yielding the same number of clusters.

This increase reflects both a modest reorganization of shared transcriptional signals and the detection of additional cluster-associated pathways under the integrated clustering, indicating that multi-view modelling reshapes RNA-level variation into more coherent functional signatures without altering the underlying expression data. To illustrate the coordinated biological organization recovered across layers, Fig. 6, available as supplementary data at *Bioinformatics Advances* online, shows the binary presence of significant GO Generic Slim Biological Process categories across RNA up-regulated markers, TF regulon activity and metabolic flux enrichments for each cluster. Categories supported concordantly in at least two layers are highlighted, providing a cluster-level summary of cross-layer functional coherence.

### 3.6 Cross-subtype validation

Application to Triple-Negative, Luminal A and Luminal B subtypes yielded qualitatively similar gains in latent-space structure and cluster interpretability (Supplementary Information, available as supplementary data at *Bioinformatics Advances* online). Although subtype-specific biological programs differed, multi-view latent embeddings generally produced clearer and more interpretable cluster structures than RNA-only analyses. This suggests that the added functional resolution arises from combining multiple inferred views rather than from subtype-specific patterns in any single layer.

## 4 Discussion

This study investigated whether functional layers inferred from scRNA-seq, capturing regulatory and metabolic activity, can be jointly modelled to improve the functional interpretation of cellular heterogeneity beyond gene expression alone. Rather than introducing new experimental modalities, we organized transcriptome-derived signals into complementary functional views and used multi-view latent factor modelling to structure their coordinated variation at single-cell resolution.

A key aspect of this work is the treatment of transcriptome-derived functional inferences as structured, biologically constrained representations, rather than as post hoc annotations appended to gene expression. Latent factor models such as MOFA+ were originally developed to integrate experimentally distinct data modalities; here, we repurpose the same formalism to organize multiple functional projections inferred from a single transcriptomic measurement within a shared latent space. In this setting, the objective is not data fusion, but the identification of coordinated regulatory and metabolic programs whose variation can be represented along interpretable low-dimensional axes.

Across analyses, multi-view modelling did not primarily increase cluster granularity, but reshaped the cellular manifold into functional dimensions supported across regulatory and metabolic layers. While major biological subdivisions were already detectable from RNA alone, the joint model aligned transcriptional variation with inferred regulatory and metabolic signals, resulting in clearer separation of non-proliferative states and more coherent functional signatures.

Comparison with transcriptome-only representation learning approaches such as scVI indicated that deep generative models can provide highly stable embeddings of transcriptional variation, but tend to organize cells primarily along dominant transcriptional gradients. In contrast, the multi-view latent representation captured coordinated regulatory and metabolic programs that were less clearly resolved in transcriptome-only embeddings. These results suggest a trade-off between partition robustness, which favours transcriptome-optimized generative models, and functional organization, which benefits from the integration of complementary regulatory and metabolic views. Methods based on weighted nearest-neighbour graph construction, such as Seurat WNN (Hao *et al.* 2021), were considered but not retained as primary comparisons because they are designed for experimentally independent modalities rather than continuous transcriptome-derived functional views. In preliminary exploratory analyses, this strategy did not improve the recovery of shared functional organization in our setting.

By modelling inferred regulon activity, metabolite-level features and metabolic fluxes as complementary projections of the transcriptome, the organizational strategy revealed patterns that were less clearly organized when each layer was analysed independently. In the HER2+ breast cancer case study, the resulting latent space highlighted proliferative, oxidative-metabolic and stress-adaptive programs as coordinated axes of variation. These programs are consistent with established features of HER2+ breast cancer biology, supporting the biological plausibility of the recovered organization without requiring additional experimental modalities.

In this framework, MOFA+ serves as an organizational latent factor model that structures multiple functionally constrained projections of the transcriptome, rather than integrating independent experimental data types. Each functional layer is treated as a distinct view with its own variance structure, allowing both shared and view-specific patterns to emerge. The resulting latent representation should therefore be interpreted as a constrained reparameterization of transcriptome-derived variability that improves functional coherence and interpretability, rather than as an increase in molecular information content.

### 4.1 Clustering robustness and quantitative validation

Clustering robustness was evaluated quantitatively through repeated 80% cell subsampling, quantified by the adjusted Rand index (ARI =0.586±0.062), demonstrating that the multi-view latent space provides stable partitions of cellular states across perturbations in cell composition. While scVI yielded higher absolute clustering stability (ARI =0.766±0.060), reflecting its strength as a generative model optimized for transcriptional signal separation, the multi-view MOFA+ representation achieved higher pathway cluster-specificity (0.784 vs. 0.734 for scVI and 0.708 for RNA-only) and recovered the largest number of significant cluster-associated RNA GO Biological Process terms (583 vs. 380 for scVI and 394 for RNA-only; Table 5, available as supplementary data at *Bioinformatics Advances* online). These results reflect a trade-off between partition robustness and functional organization, rather than a simple ranking of methods by a single metric.

### 4.2 Cross-view concordance and biological structure

Quantitative analysis of cross-view concordance through RV coefficient analysis revealed that regulatory activity strongly covaries with RNA expression (RV =0.75), while metabolic views display moderate but significant association (RV ranging from 0.24 to 0.42; Fig. 5, available as supplementary data at *Bioinformatics Advances* online). Importantly, after residualization of the dominant RNA component, coordinated residual structure persists among functional views (TF-metabolite RV =0.55; TF-flux RV =0.59; metabolite-flux RV =0.75) (Robert and Escoufier 1976). This residual concordance should not be interpreted as evidence of independent molecular information, but rather as structure induced by distinct biological constraints embedded in the inference models used to derive each functional layer. These results support the interpretation that regulatory and metabolic layers capture distinct yet coordinated biological constraints beyond the dominant transcriptomic signal, supporting the biological relevance of the multi-view integration.

Several limitations arise from the inferred nature of the functional layers. Methods such as pySCENIC and scFEA rely on modelling assumptions, prior knowledge and parameter choices, and alternative inference strategies may lead to partially different functional representations. Because the functional views used in this framework are computationally inferred, biases, dropouts, or limitations inherent to the upstream inference algorithms may propagate into the MOFA+ latent representation and should therefore be considered when interpreting the resulting functional organization.

Accordingly, the aim of this work is not to validate individual inference methods, nor to approximate multi-omic measurements, but to assess whether multiple transcriptome-derived functional projections converge onto a coherent latent organization when considered jointly. As with other latent factor approaches, the specific structure of the latent space depends on modelling choices, including the number of factors and downstream analysis parameters, which were selected based on convergence and interpretability. The qualitatively consistent improvements in latent-space organization and cluster interpretability observed across four molecularly distinct breast cancer subtypes suggest that the overall organizational structure is robust to dataset-specific variation, even if individual pathway assignments may differ under alternative parameter settings or inference methods.

Future work could evaluate this organizational strategy on datasets where matched transcriptomic, regulatory and metabolic measurements are experimentally available, providing an orthogonal validation of the inferred functional representations.

Within this scope, we observed qualitatively similar improvements in latent-space structure and cluster interpretability across multiple breast cancer subtypes, supporting applicability beyond a single dataset or cell-line composition. More broadly, functional multi-view modelling provides an organizational layer between gene expression and downstream biological interpretation, complementing standard scRNA-seq analyses in contexts where matched multi-omic measurements are not available. By positioning cells in a shared latent space informed by multiple transcriptome-derived functional representations, this approach enables a more informative characterization of cellular states, in which regulatory and metabolic dimensions jointly contribute to the organization of cellular heterogeneity.

## 5 Conclusion

This work demonstrates that multiple functional layers inferred from scRNA-seq can be jointly modelled to organize cellular heterogeneity into an interpretable latent representation. By integrating transcriptomic, regulatory and metabolic projections within a shared latent space, the proposed organizational strategy restructures RNA-derived variation into coordinated functional programs that extend beyond gene expression alone.

Although these layers are computational proxies, their joint organization provides a practical and extensible strategy for improving functional characterization from scRNA-seq in the absence of matched multi-omic profiling, while remaining fully compatible with standard single-cell analysis workflows.

## Supplementary Material

vbag185_Supplementary_Data

## Data Availability

The data underlying this article were derived from sources in the public domain: the single-cell RNA-seq dataset published by Gambardella et al. (2022), available in the Gene Expression Omnibus (GEO) repository under accession code GSE173634.
