# Supplementary material for: A latent factor framework to organize regulatory and metabolic programs inferred from scRNA-seq
Source: Bioinform Adv. 2026 Jun 30;6(1):vbag185. doi: 10.1093/bioadv/vbag185 (PMC13412163; doi:10.1093/bioadv/vbag185)
Supplement: vbag185_Supplementary_Data [file vbag185_supplementary_data.pdf]

Supplementary Information

A latent factor framework to organize regulatory and metabolic programs inferred from scRNA-seq

Chiara Napoli[1,2,*], Francesco Bardozzo[1,3], Suraj Verma[4], Le Minh Thao Doan[4], Pierpaolo Fiore[1], Carmen Faggiano[1], Claudio Angione[4], Annalisa Occhipinti[4], and Roberto Tagliaferri[1]

[1]NeuroneLab - Department of Management and Innovation Systems (DISA-MIS), University of Salerno, Italy
[2]Department of Biochemical Sciences "A. Rossi Fanelli", Sapienza University of Rome, Italy
[3]NAIR Center, Pamplona, Spain
[4]School of Computing, Engineering and Digital Technologies, Teesside University, UK

S1 Supplementary Methods and Figures

S1.1 Additional analysis on HER2+

S1.1.1 Elbow plot for factor selection

The number of latent factors was determined by examining the variance explained across all four transcriptome-derived functional views for successive MOFA+ factors. As shown in Supplementary Figure S1, the variance explained decreases progressively across factors, with the marginal contribution falling below 5% from factor 7 onwards and reaching 1.4% at factor 10. This indicates diminishing returns from additional factors beyond this point. We selected a 10-factor model as the optimal balance between capturing biological variation and maintaining interpretability of the latent space. The numerical values underlying this figure are reported in Supplementary Table S6.

S1.2 Transcriptomic and deep-learning baselines

S1.2.1 scVI as a deep-learning transcriptomic baseline

To provide a stronger baseline for transcriptomic representation learning, we applied scVI to the HER2+ dataset using default hyperparameters (2 hidden layers, 128 hidden units, latent dimension = 10). Neighbourhood graph construction, BBKNN batch correction, UMAP visualization, and Leiden clustering (resolution = 0.6) were performed on the scVI latent embedding following the same downstream pipeline used for MOFA+. Cluster markers and GO BP enrichment were computed on the RNA layer as described in the Methods section.

As shown in Supplementary Fig. S3, scVI yields a compact and stable transcriptomic partition dominated by proliferative programs. Quantitatively, scVI achieves higher clustering stability under repeated 80% cell subsampling (ARI = 0.766 ± 0.060) compared to MOFA+ (ARI = 0.586 ± 0.062), reflecting its strength as a generative model optimized for transcriptomic signal separation. However, MOFA+ recovers a larger number of cluster-associated GO terms (583 vs 380) and higher pathway cluster-specificity (0.784 vs 0.734). We interpret this as a trade-off between partition robustness and functional coherence rather than a ranking of overall performance.

S1.2.2 Early-fusion baseline via concatenation and PCA

As an early-fusion multi-view baseline, we concatenated cell-matched feature matrices across all transcriptome-derived functional views (RNA expression, TF regulon activities, inferred metabolite-level features, and predicted metabolic fluxes). Features were standardized across cells (z-scoring), and dimensionality reduction was performed by principal component analysis (PCA; 30 components; scikit-learn). A shared neighbourhood graph was constructed from the PCA embedding using BBKNN (batch key: cell line; 5 neighbours within batch), followed by 3D UMAP visualization and Leiden clustering (resolution = 0.6) in Scanpy.

Cluster markers were identified on the RNA layer using Wilcoxon rank-sum tests computed on the full gene set stored in `.raw` (adjusted $p < 0.05$, logFC > 0.5). Functional annotation was performed by Gene Ontology Biological Process (GO BP) enrichment using Enrichr (FDR < 0.05). Under this baseline, the dominant clustering structure was largely aligned with cell-cycle–associated variation, with limited additional resolution of non-proliferative functional states (Supplementary Fig. S2).

S1.2.3 2D UMAP comparison across methods

To complement the 3D visualizations in the main text and facilitate direct visual comparison across methods, Supplementary Figure S4 shows 2D UMAP projections for the RNA-only baseline, scVI, and MOFA+ embeddings, both before (coloured by cell line) and after BBKNN correction (coloured by Leiden cluster). The pre-BBKNN panels illustrate the degree of cell-line separation in each latent space, while the post-BBKNN panels show the resulting cluster structure after neighbourhood harmonization.

S1.3 Cross-view concordance of inferred functional layers

To quantify the extent to which inferred functional layers share coordinated cell-wise structure, we computed pairwise cross-view similarity using the RV coefficient (Supplementary Fig. S5). For each view, PCA scores were computed on z-scored feature matrices (15 components), and RV coefficients were evaluated on sample-wise centred Gram matrices derived from these scores, enabling comparison at the level of shared cell-wise organisation rather than direct feature correspondence.

Pairwise similarity analysis revealed strong concordance between RNA expression and TF regulon activity (RV = 0.75), moderate association between RNA and metabolic fluxes (RV = 0.42), and weaker similarity between RNA and metabolite-level features (RV = 0.24). In contrast, the two metabolic layers exhibited substantial mutual concordance (metabolites–fluxes RV = 0.66), indicating internally coherent metabolic organisation across cells.

To assess cross-view similarity beyond dominant transcriptomic structure, we repeated the analysis after residualizing non-RNA views with respect to RNA principal components explaining 90% of transcriptomic variance. RNA-related similarity was substantially reduced, while concordance persisted among functional layers (TF–flux RV = 0.59; TF–metabolites RV = 0.55; metabolites–fluxes RV = 0.75). This residual structure is consistent with coordinated organisation across inferred functional projections beyond the dominant RNA-associated component.

S1.4 Cross-layer biological concordance per cluster

To illustrate the coordinated regulatory and metabolic organization recovered by the multi-view framework, we visualized binary GO Generic Slim Biological Process enrichment (FDR < 0.05) across three functional layers, RNA up-regulated markers, TF regulon activity, and metabolic flux, for each cluster identified in the HER2+ dataset (Supplementary Fig. S6). Terms supported concordantly in at least two layers are highlighted in yellow, providing an illustrative cluster-level summary of cross-layer functional organization.

S1.5 Cluster composition in the HER2+ dataset

Tables S1–S3 report the number of cells per cluster for RNA-only, scVI, and MOFA+ clustering respectively, providing quantitative summaries of the cluster structures illustrated in Supplementary Figure S4.

Table S1: Cluster composition under RNA-only Leiden clustering (resolution = 0.6, 6 clusters).

Cluster	n cells
0	1210
1	763
2	748
3	668
4	525
5	410
Total	4324

Table S2: Cluster composition under scVI Leiden clustering (resolution = 0.6, 4 clusters).

Cluster	n cells
0	1610
1	1610
2	1016
3	88
Total	4324

Table S3: Cluster composition under MOFA+ multi-view Leiden clustering (resolution = 0.6, 6 clusters). Biological module assignments reflect the functional interpretation described in the main text.

Cluster	n cells	Biological module
0	1049	Proliferative-biosynthetic
1	962	Proliferative-biosynthetic
2	840	Oxidative-metabolic
3	638	Oxidative-metabolic
4	625	Stress-adaptive
5	210	Oxidative-metabolic
Total	4324	

S1.6 Cell line composition in the HER2+ dataset

Table S4 reports the number of cells per cell line in the HER2+ dataset after quality control.

S1.7 Quantitative comparison with alternative methods

Table S5 reports clustering performance and RNA-layer interpretability metrics for three approaches: scRNA-only analysis, scVI, and multi-view MOFA+. While scVI achieves the highest clustering stability under subsampling, MOFA+ recovers more cluster-associated GO terms and higher pathway cluster-specificity, reflecting a trade-off between partition robustness and functional coherence across layers.

Table S4: Number of cells per cell line in the HER2+ dataset after quality control.

Cell line	n cells
HCC1954	1616
MDAMB453	1119
AU565	589
JIMT1	564
EVSAT	436
Total	4324

Table S5: Quantitative comparison of clustering performance and RNA-layer interpretability across three methods. ARI: adjusted Rand index (mean ± sd across 10 repeated 80% cell subsampling iterations). RNA specificity: fraction of significantly enriched GO BP pathways unique to a single cluster.

Method	Input	Res.	Clust.	ARI	ARI sd	Spec.	GO
scRNA-only	scRNA	0.6	6	0.524	0.077	0.708	394
scVI	scRNA	0.6	4	0.766	0.060	0.734	380
MOFA+	RNA+TF+Flux+Met	0.6	6	0.586	0.062	0.784	583

S1.8 MOFA+ variance decomposition per factor and view

Table S6 reports the variance explained by each MOFA+ latent factor across the four functional views in the HER2+ dataset, providing the numerical values underlying Supplementary Fig. S1 and main text Fig. 3.

Table S6: Variance explained (%) by each MOFA+ latent factor across functional views in the HER2+ dataset. Values represent view-specific reconstruction performance (R^2) for each factor. Total is the sum across views for each factor.

Factor	scRNA-seq	TFs	Metabolites	Fluxes	Total
Factor 1	4.88	23.79	2.89	3.69	35.25
Factor 2	3.06	8.35	1.79	5.33	18.54
Factor 3	4.11	6.81	2.58	3.89	17.39
Factor 4	0.00	2.95	7.22	6.66	16.83
Factor 5	2.10	3.51	2.29	4.92	12.81
Factor 6	0.89	7.86	0.32	0.16	9.23
Factor 7	0.03	0.09	2.56	2.40	5.07
Factor 8	0.45	3.71	0.21	0.17	4.54
Factor 9	0.46	2.23	0.11	0.07	2.88
Factor 10	0.28	0.64	0.28	0.17	1.37

S2 Additional analyses on Luminal A

The Luminal A subset comprised 7,516 cells across nine cell lines. After quality control, 2,663 HVGs were retained for the RNA baseline. The multi-view dataset included four layers—scRNA-seq expression, 245 TF-regulon activities, 70 metabolite-imbalance features, and 168 flux reactions—integrated using a MOFA+ model with 10 latent factors.

In the RNA-only embedding (Fig. S7A–B), transcriptional variation was dominated by a proliferative gradient, with cluster boundaries appearing broad and partially overlapping. RNA-

level GO enrichments highlighted proliferation- and cell-cycle–related processes that were shared across multiple clusters.

The integrated embedding (Fig. S7C–F) recovered the same cluster structure but aligned transcriptional signals with regulatory and metabolic features. Proliferative clusters showed coherent activation of E2F-family regulons, DNA-repair pathways and glycolytic or pyruvate-associated fluxes, while non-proliferative clusters were distinguished by lipid, steroid and monocarboxylate metabolic programs.

Overall, in Luminal A, integrated clustering markedly increased the cluster-specificity of upregulated RNA pathways, resolving functionally coherent proliferative and metabolic programs that were only partially defined in the RNA-only baseline.

S3 Additional analyses on Luminal B

The Luminal B subset comprised 2,908 cells across two cell lines. After filtering, 2,397 HVGs were selected. The four-view dataset—RNA expression, 209 TF-regulon activities, 70 metabolite features and 168 flux reactions—was integrated through a MOFA+ model with ten latent factors. Leiden clustering was applied at resolution 0.4 for both embeddings.

In the RNA-only embedding (Fig. S8A–B), transcriptional variation was largely driven by cell-cycle and DNA maintenance programs, with additional contributions from translational and oxidative metabolic processes.

Following integration (Fig. S8C–F), a comparable cluster partition was obtained, but functional signals appeared more coherently aligned across transcriptional, regulatory and metabolic layers. Clusters enriched for cell-cycle/DNA programs showed concordant regulatory activation together with oxidative phosphorylation and amino-acid–related metabolic signatures, while other clusters were distinguished by regulatory programs linked to transcriptional control and autophagy-related processes.

Overall, in Luminal B, integrated modelling modestly increased the cluster-specificity of upregulated RNA pathways (0.66 vs. 0.58) by reinforcing the functional coherence of transcriptional programs across regulatory and metabolic views.

S4 Additional analyses on Triple-Negative

S4.1 TNBC-A

The TNBC-A subset comprised 11,679 cells across eight cell lines. After filtering, 2,726 highly variable genes were retained. RNA expression, 233 TF-regulon activities, 70 metabolite features and 168 flux reactions were integrated using a MOFA+ model with ten latent factors. Leiden clustering at resolution 0.4 produced five clusters in both the RNA-only and integrated embeddings.

In the RNA-only embedding (Fig. S9A–B), RNA-level GO signatures were strongly cluster-associated, with high pathway cluster-specificity for both up- and down-regulated terms.

Following integration (Fig. S9C–F), the same clustering granularity was retained, but RNA-level functional programs became more broadly shared across clusters. Consistent with this, RNA pathway cluster-specificity decreased in the integrated clustering (up: 0.78 vs. 0.91), reflecting the emergence of shared proliferative and metabolic axes supported by concordant regulatory and metabolic signals rather than strictly cluster-restricted RNA programs.

Overall, in TNBC-A, multi-view modelling highlighted a highly interconnected transcriptional landscape in which major functional programs span multiple clusters, reducing apparent RNA-only cluster exclusivity.

S4.2 TNBC-B

The TNBC-B subset included 6,954 cells across seven cell lines. RNA expression, TF-regulon activities, metabolite features and flux reactions were integrated using the same MOFA+ configuration adopted for the other subtypes. Seven clusters were identified in both the RNA-only and integrated embeddings.

In the RNA-only embedding (Fig. S10A–B), RNA-level GO programs were partially shared across clusters, consistent with moderate pathway overlap between functional groups.

Following integration (Fig. S10C–F), the same cluster structure was preserved, but RNA-level functional programs became more clearly cluster-associated. RNA pathway cluster-specificity increased under the integrated clustering for both up- and down-regulated terms (up: 0.82 vs. 0.75; down: 0.82 vs. 0.63), accompanied by reduced cross-cluster sharing (mean clusters per term: up 1.21 vs. 1.27; down 1.22 vs. 1.59).

Overall, in TNBC-B, multi-view modelling redistributed transcriptional signals into more distinct functional signatures supported by concordant regulatory and metabolic features, improving RNA-level interpretability relative to the RNA-only baseline.

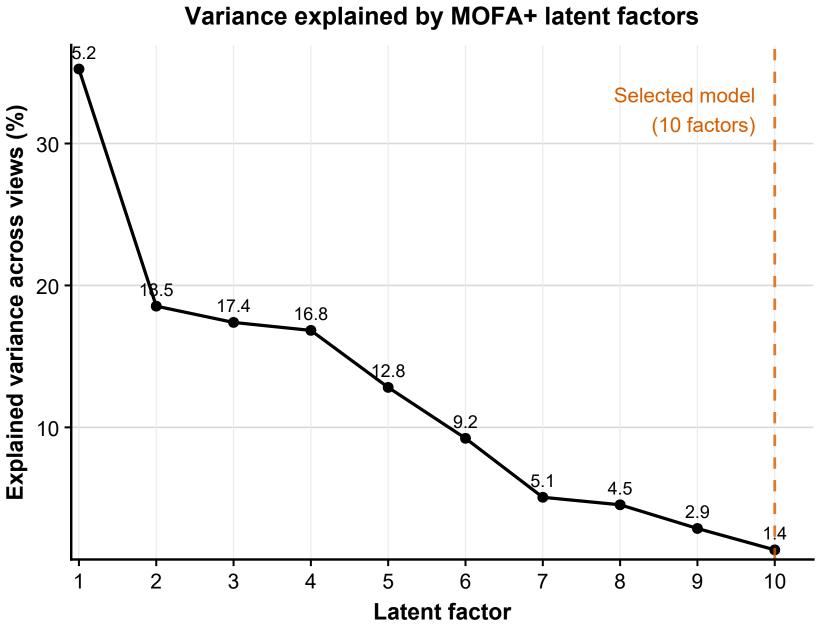

Figure S1: Variance explained by successive MOFA+ latent factors across the four functional views. The marginal contribution falls below 5% from factor 7 onwards and reaches 1.4% at factor 10 (orange dashed line), indicating diminishing returns from additional factors and supporting the selection of a 10-factor model. Numerical values are reported in Supplementary Table S6.

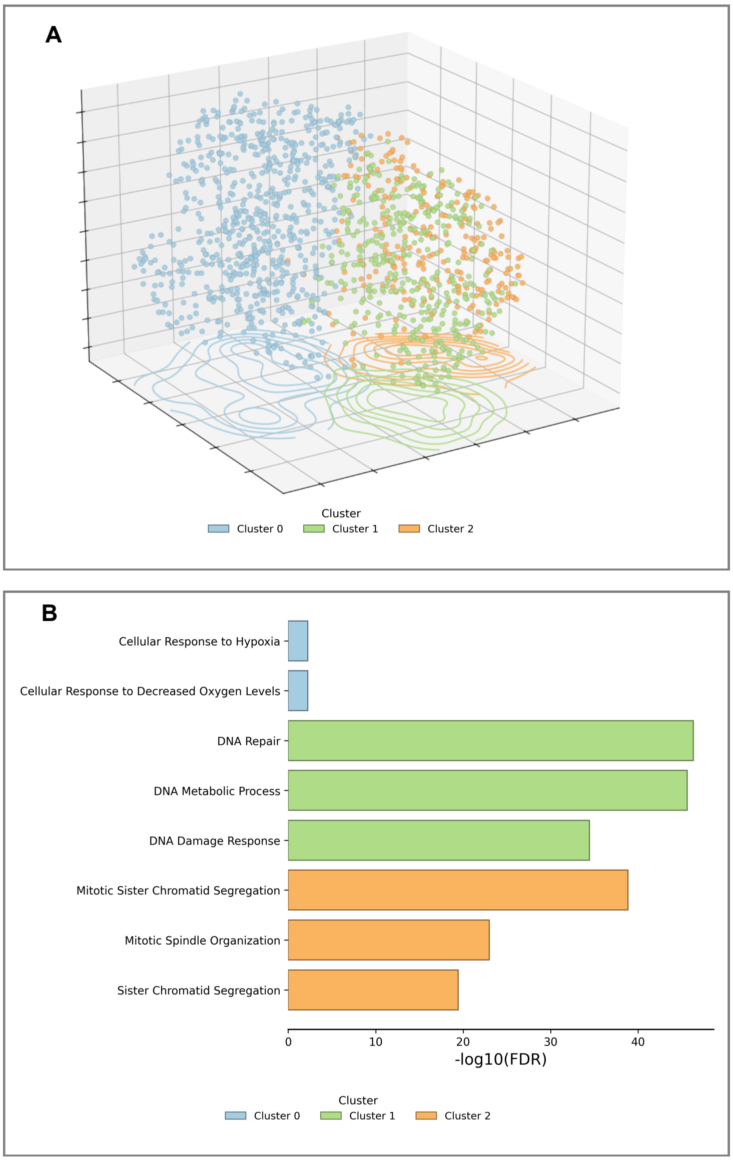

Figure S2: **Early-fusion PCA baseline is dominated by proliferation-associated structure. (A)** 3D UMAP of concatenated multi-view features after PCA and BBKNN graph construction, coloured by Leiden clusters (resolution = 0.6). Density iso-contours indicate cluster distributions on the base plane. For visualization only, a random 30% subsampling of cells was applied to reduce overplotting. **(B)** Top GO BP terms enriched among up-regulated RNA markers for each cluster (Enrichr; $-\log_{10}$FDR). The resulting clustering is primarily aligned with cell-cycle and DNA maintenance programs, with limited additional resolution of non-proliferative functional states.

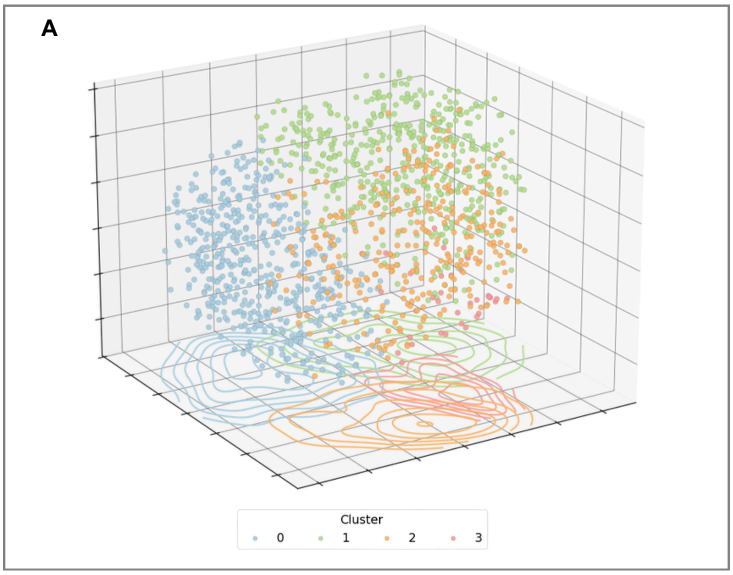

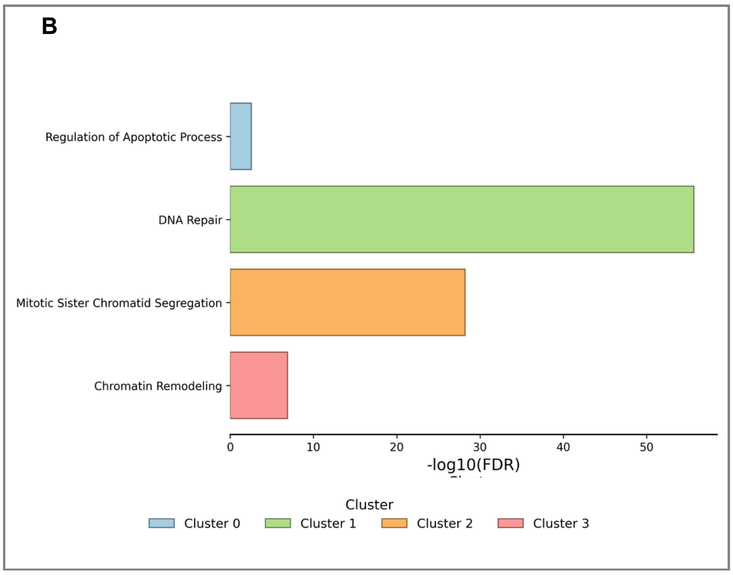

Figure S3: **scVI as a deep-learning transcriptomic baseline. (A)** 3D UMAP of the scVI latent embedding after BBKNN graph construction, coloured by Leiden clusters (resolution = 0.6). Density iso-contours indicate cluster distributions on the base plane. For visualization only, a random 30% subsampling of cells was applied to reduce overplotting. **(B)** Top GO BP terms enriched among up-regulated RNA markers for each cluster (Enrichr; $-\log_{10}$FDR). The resulting clustering is primarily aligned with proliferative and DNA-maintenance programs, with less extensive recovery of additional cluster-associated RNA functional states than the multi-view MOFA+ framework.

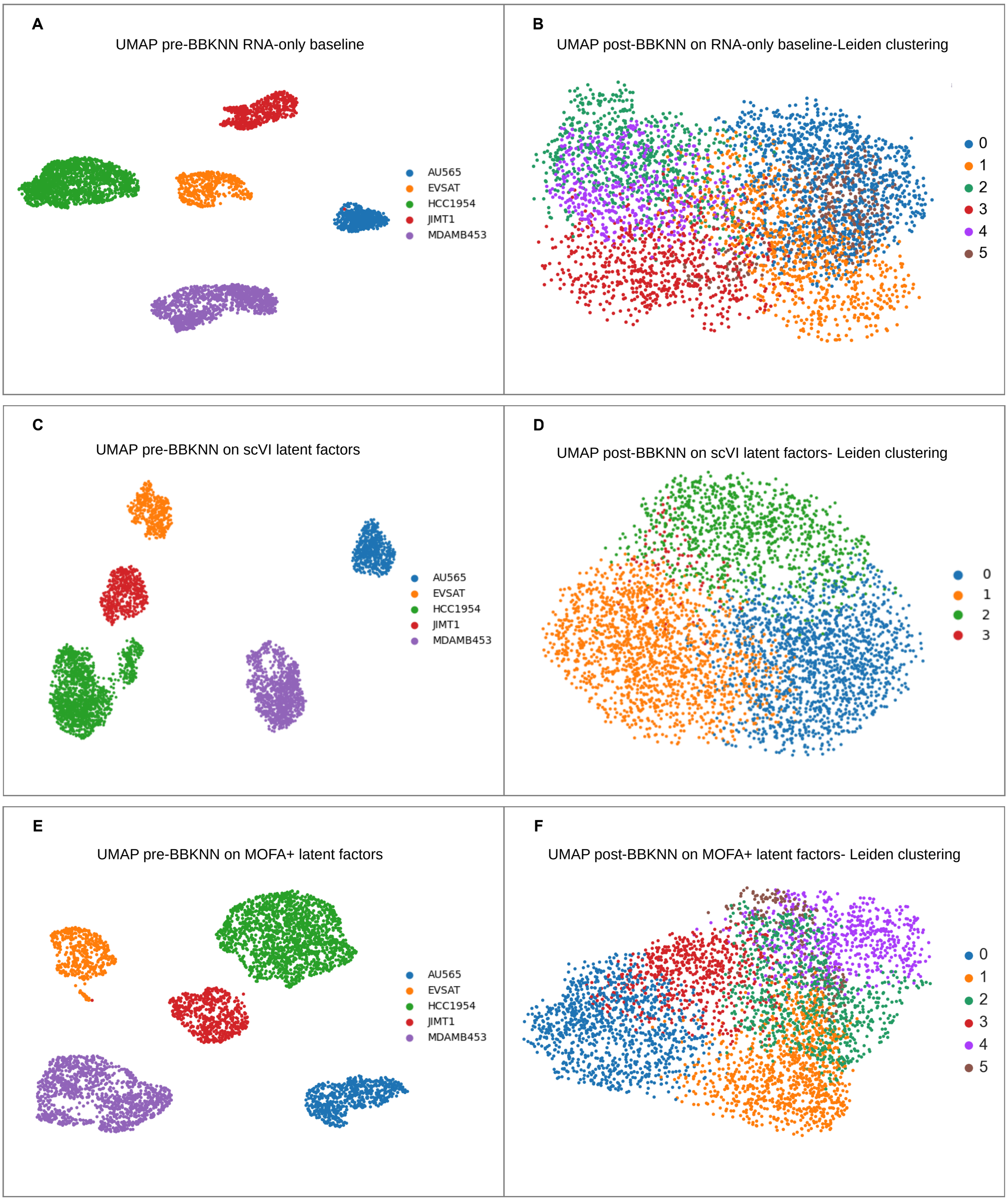

Figure S4: **2D UMAP comparison of RNA-only, scVI and MOFA+ embeddings before and after BBKNN correction.** **(A)** RNA-only embedding coloured by cell line (pre-BBKNN). **(B)** RNA-only embedding coloured by Leiden cluster (post-BBKNN). **(C)** scVI embedding coloured by cell line (pre-BBKNN). **(D)** scVI embedding coloured by Leiden cluster (post-BBKNN). **(E)** MOFA+ embedding coloured by cell line (pre-BBKNN). **(F)** MOFA+ embedding coloured by Leiden cluster (post-BBKNN). All embeddings were computed on the full cell set without subsampling.

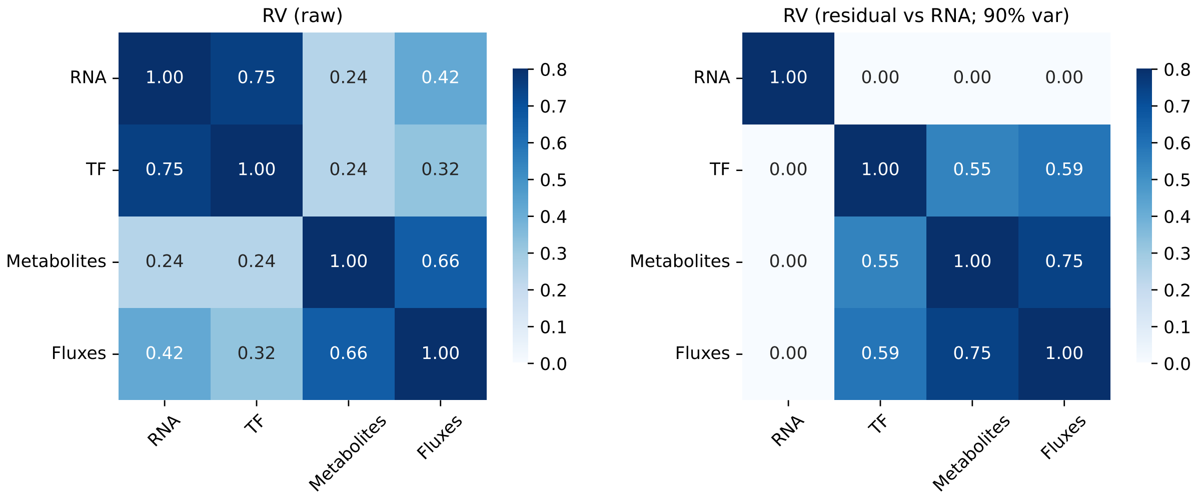

Figure S5: **Cross-view similarity between inferred functional layers before and after residualization of dominant transcriptomic variation.** Pairwise RV coefficients were computed on PCA scores from RNA, TF regulon activity, metabolite-level features and metabolic fluxes across repeated subsampling. (A) Raw cross-view similarity. (B) Similarity after residualization with respect to RNA principal components explaining 90% of transcriptomic variance. RNA-related similarity is effectively removed, whereas substantial concordance persists among functional layers, particularly between metabolic projections, indicating coordinated structure beyond dominant transcriptomic variation.

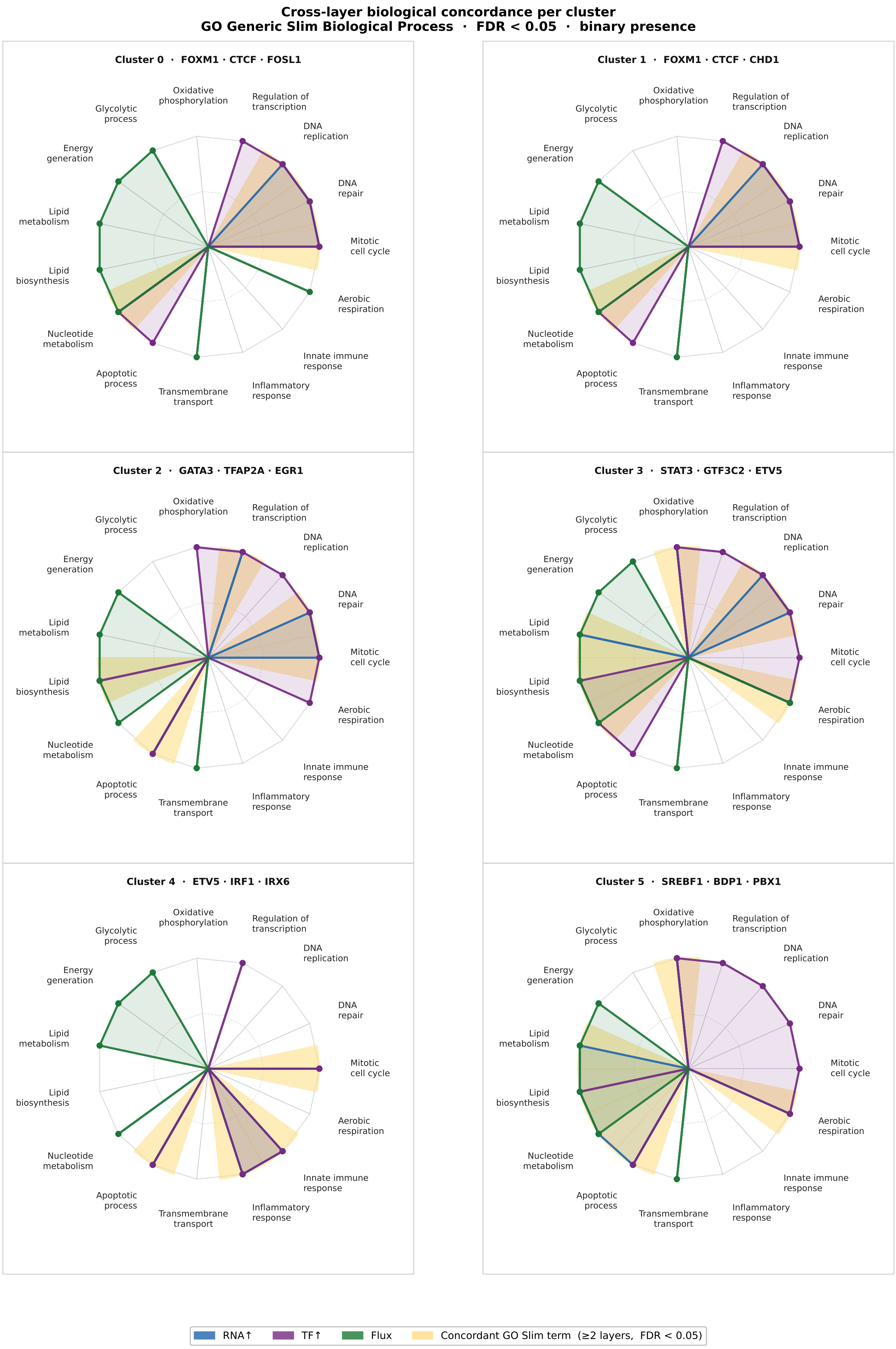

Figure S6: **Illustrative summary of cross-layer biological concordance across HER2+ clusters.** For each MOFA+ derived cluster, radar plots show the binary presence of significant GO Generic Slim Biological Process categories (FDR < 0.05) across RNA up-regulated markers, TF regulon activity, and metabolic flux enrichments. Yellow shading highlights categories supported by at least two layers within the same cluster.

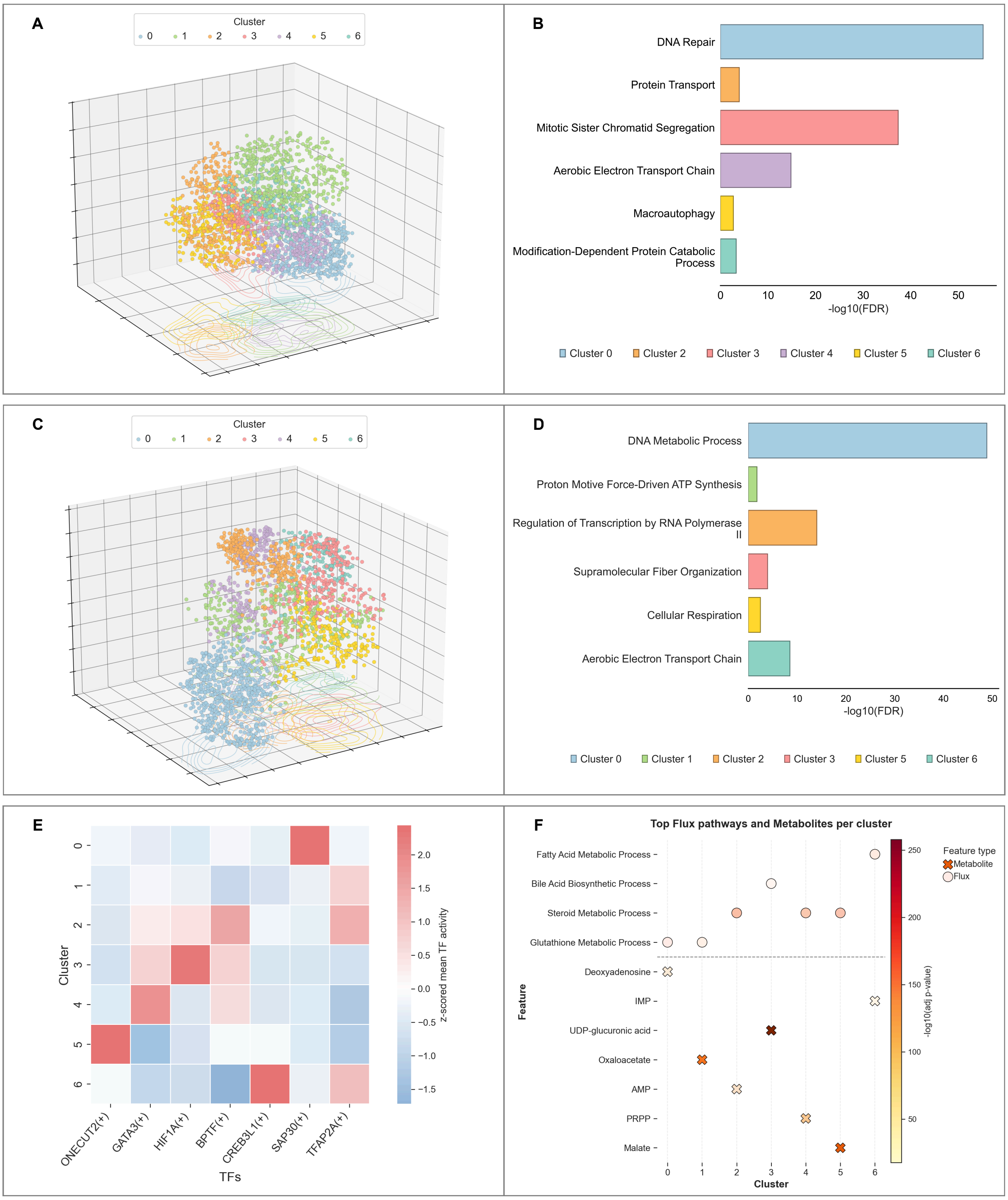

Figure S7: **Luminal A multi-view analysis.** (A) 3D UMAP of MOFA+ latent space (30% subsample for visualization). (B) RNA-level GO term enrichments for up-regulated genes. (C) 3D UMAP after BBKNN correction (30% subsample for visualization). (D) GO enrichments under the integrated clustering. (E) TF-regulon activity heatmap. (F) Significant metabolite imbalance and flux markers per cluster.

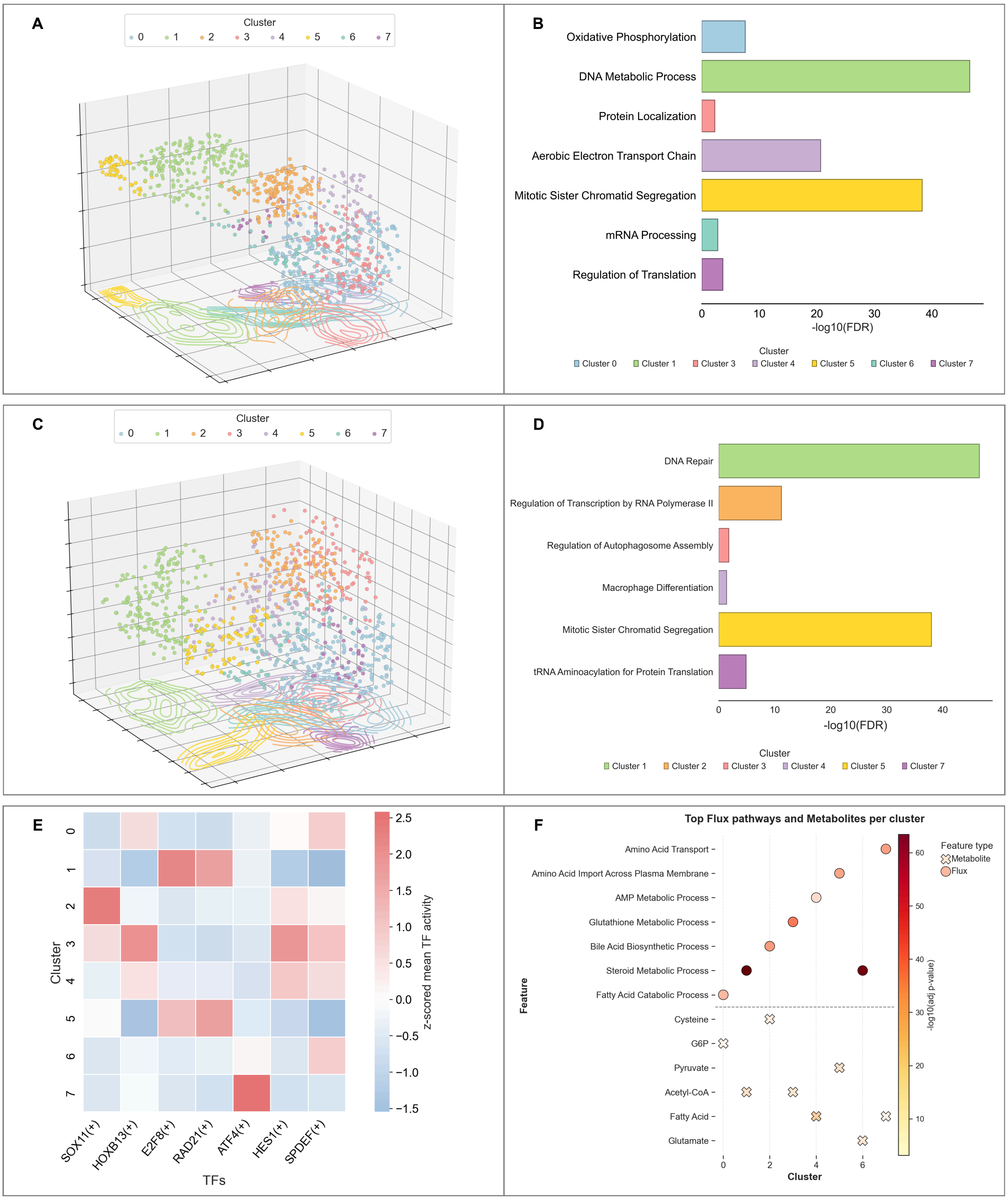

Figure S8: **Luminal B multi-view analysis.** (A) 3D UMAP of MOFA+ latent space (30% subsample for visualization). (B) RNA-level GO term enrichments for up-regulated genes. (C) 3D UMAP after BBKNN correction (30% subsample for visualization). (D) GO enrichments under the integrated clustering. (E) TF-regulon activity heatmap. (F) Significant metabolite imbalance and flux markers per cluster.

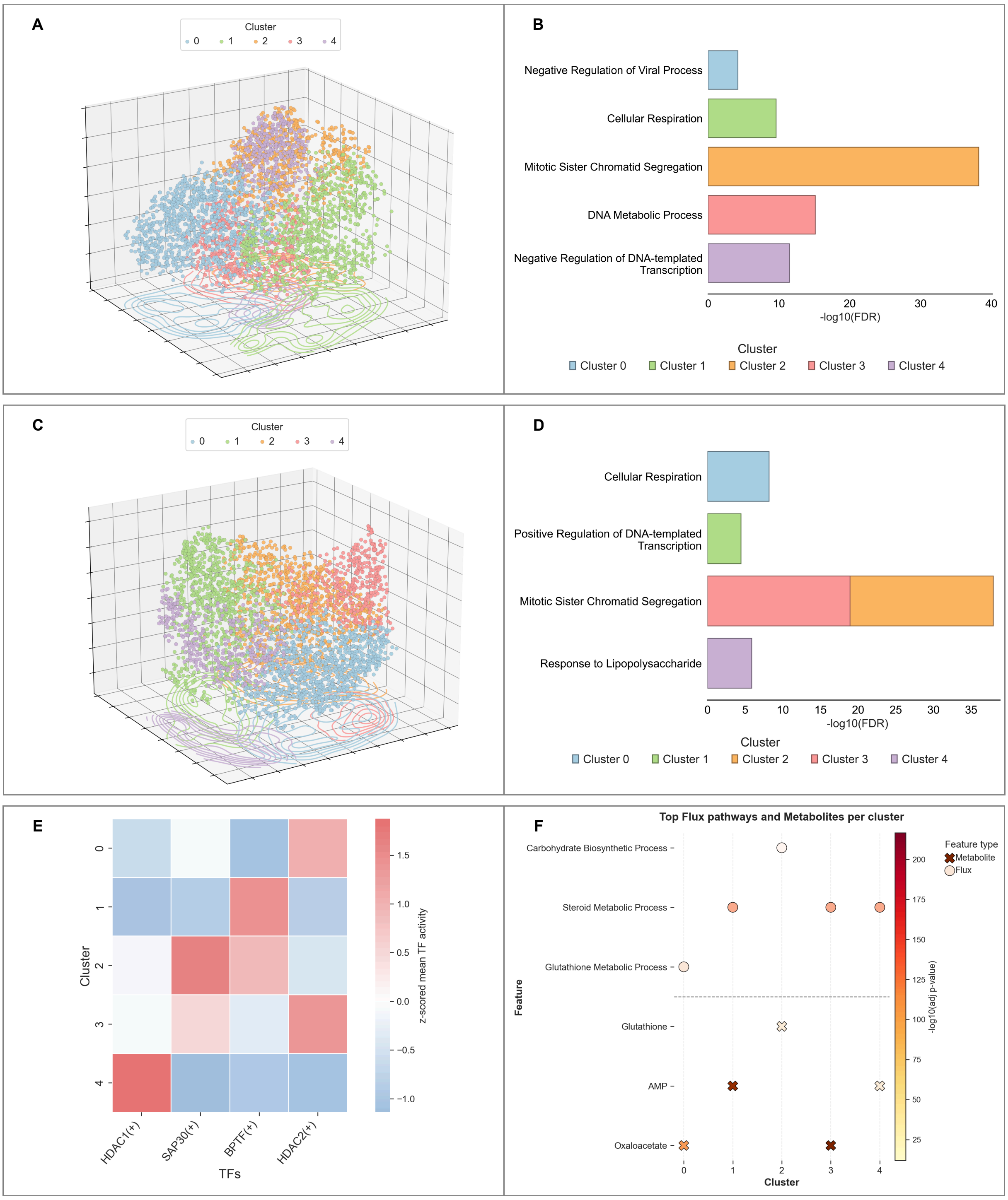

Figure S9: **TNBC-A multi-view analysis.** (A) 3D UMAP of MOFA+ latent space (30% subsample for visualization). (B) RNA-level GO term enrichments for up-regulated genes. (C) 3D UMAP after BBKNN correction (30% subsample for visualization). (D) GO enrichments under the integrated clustering. (E) TF-regulon activity heatmap. (F) Significant metabolite imbalance and flux markers per cluster.

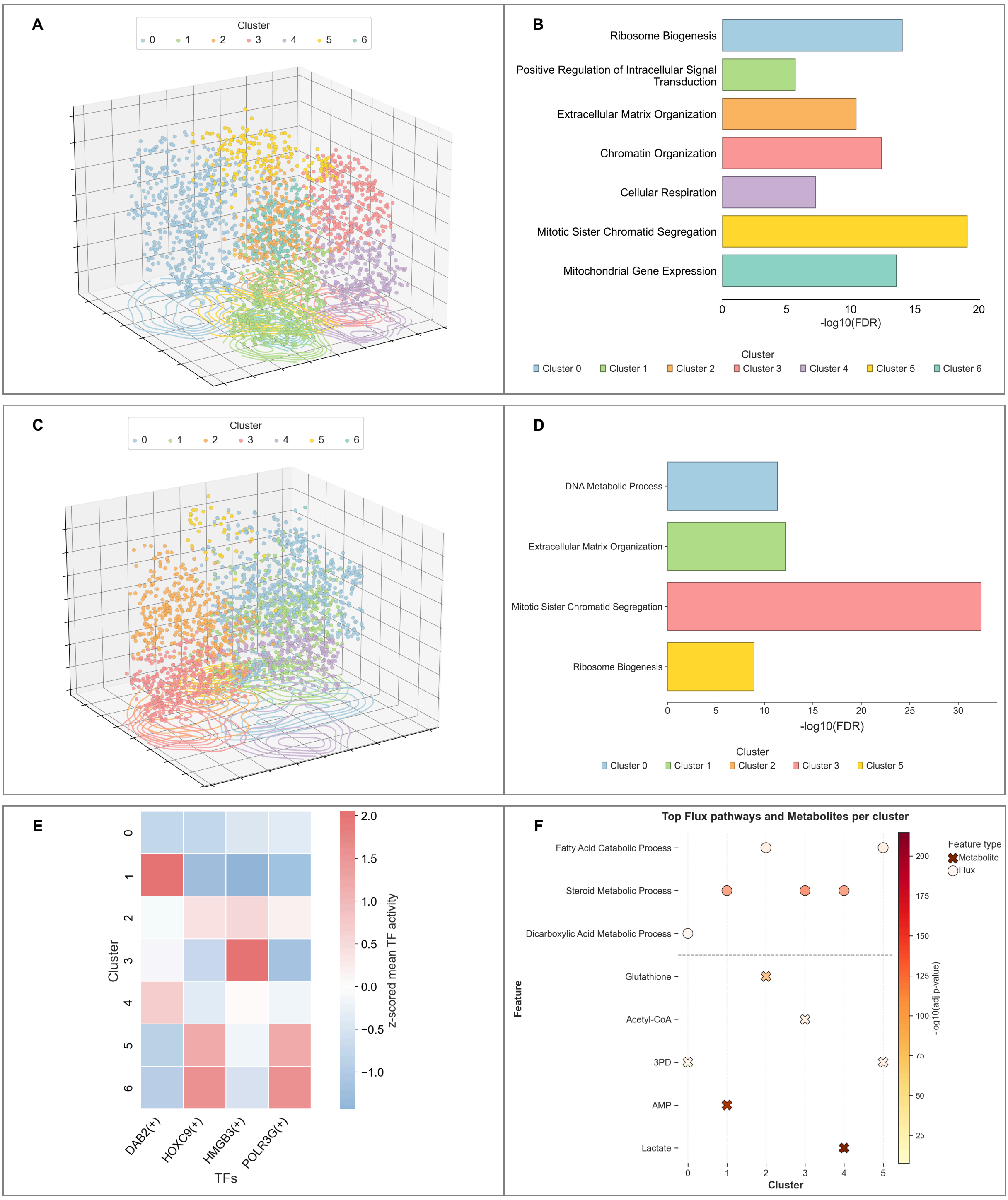

Figure S10: **TNBC-B multi-view analysis.** (A) 3D UMAP of MOFA+ latent space (30% subsample for visualization). (B) RNA-level GO term enrichments for up-regulated genes. (C) 3D UMAP after BBKNN correction (30% subsample for visualization). (D) GO enrichments under the integrated clustering. (E) TF-regulon activity heatmap. (F) Significant metabolite imbalance and flux markers per cluster.
